# EUS-Guided Shear-Wave-Based Elastography: Current Evidence and the Emerging Role of Two-Dimensional Shear-Wave Elastography

**DOI:** 10.3390/diagnostics16162505

**Published:** 2026-08-08

**Authors:** Andrea Lisotti, Yasunobu Yamashita, Emilija Rakichevikj, Graziella Masciangelo, Antonio Fuso, Pasquale Dragone, Simona Guglielmo, Maria Cristina D’Ercole, Rosa Federica La Fortezza, Masayuki Kitano, Pietro Fusaroli

**Affiliations:** 1 Gastroenterology Unit, Hospital of Imola, University of Bologna, 40026 Imola, BO, Italy; emilijarakichevikj.md@gmail.com (E.R.); masciangelo.lm@gmail.com (G.M.); antoniofuso96@libero.it (A.F.); pasquale.dragone93@gmail.com (P.D.); guglielmosimona@gmail.com (S.G.); mcristinadercole@gmail.com (M.C.D.); rf.lafortezza@gmail.com (R.F.L.F.); pietro.fusaroli@unibo.it (P.F.); 2 Second Department of Internal Medicine, Wakayama Medical University, Wakayama 641-0012, Japan; yasunobu@wakayama-med.ac.jp (Y.Y.); kitano@wakayama-med.ac.jp (M.K.)

**Keywords:** endoscopic ultrasound, two-dimensional shear-wave elastography, EUS-SWE, pancreatic elastography, chronic pancreatitis, autoimmune pancreatitis, pancreatic cancer, liver fibrosis, endo-hepatology, stiffness, shear-wave elastography

## Abstract

Elastography is an ultrasound-based technique that enables the non-invasive assessment of tissue stiffness. In endoscopic ultrasound (EUS), elastography was initially introduced as a strain-based method, providing qualitative or semi-quantitative information on tissue deformation. More recent technological developments have enabled the integration of shear-wave-based elastography into EUS platforms, including shear-wave measurement (SWM), point shear-wave elastography (pSWE), and two-dimensional shear-wave elastography (2D-SWE). Unlike strain elastography, shear-wave techniques provide quantitative estimates of tissue stiffness by measuring shear-wave velocity or a derived elastic modulus. This invited narrative review summarizes the technical principles, terminology, quality-control requirements, and current clinical evidence for EUS-guided shear-wave-based elastography in pancreatic and hepatobiliary diseases. Because much of the available EUS literature derives from EUS-SWM or point SWE rather than true 2D-SWE, acquisition mode is a central determinant when interpreting clinical evidence and clinical readiness. Current data suggest that EUS-guided shear-wave-based elastography is technically feasible and biologically plausible, but its clinical maturity remains indication-specific. Evidence is most encouraging in chronic pancreatitis, early chronic pancreatitis, autoimmune pancreatitis activity monitoring, and selected endo-hepatology settings, particularly liver fibrosis assessment in patients in whom transabdominal techniques may be suboptimal. In contrast, available evidence does not support EUS-guided shear-wave-based elastography as a standalone diagnostic test for differentiating solid pancreatic lesions, including pancreatic ductal adenocarcinoma, because absolute stiffness values often overlap among malignant lesions, inflammatory masses, and background parenchyma. Before routine clinical implementation, standardized acquisition protocols, disease-specific cut-offs, multicenter reproducibility data, and evidence of incremental clinical value beyond established diagnostic pathways are required.

## 1. Introduction

Elastography is an ultrasound-based imaging technique that allows the non-invasive assessment of tissue stiffness. Elastographic methods have attracted substantial scientific and clinical interest because many pathological processes alter the mechanical properties of tissues. Fibrosis, inflammation, edema, necrosis, and malignant infiltration may all produce measurable changes in tissue elasticity. In gastroenterology and pancreatobiliary imaging, these mechanical changes are particularly relevant because several clinically important diseases, including chronic pancreatitis, autoimmune pancreatitis, pancreatic ductal adenocarcinoma, liver fibrosis, and portal hypertension, are characterized by variable degrees of tissue remodeling [1,2].

Endoscopic ultrasound (EUS) elastography was initially introduced as a strain-based technique, providing qualitative or semi-quantitative assessment of tissue stiffness through color-coded maps, strain ratios, or histogram-based parameters. Strain elastography has been explored mainly for focal pancreatic lesions and lymph nodes, where malignant tissue tends to deform less than surrounding tissue. However, strain elastography remains intrinsically comparative, compression-dependent, and operator-sensitive. It does not directly measure tissue stiffness, and its reproducibility may be limited by differences in scope position, cardiac and respiratory motion, and the degree of tissue compression applied during acquisition [3,4].

More recently, technological developments have enabled the integration of shear-wave-based elastography into EUS platforms. Unlike strain elastography, shear-wave elastography (SWE) provides quantitative measurements by analyzing the propagation velocity of shear-waves generated within the tissue. The availability of EUS-guided shear-wave measurement (EUS-SWM), point SWE, and two-dimensional SWE has created new opportunities for objective assessment of pancreatic and hepatobiliary diseases, especially when the target organ is deep, difficult to access transabdominally, or requires concomitant EUS-guided tissue acquisition or hemodynamic assessment [5,6,7].

The clinical question, however, is not whether EUS-guided shear-wave-based elastography can measure stiffness, but whether these measurements are sufficiently reliable, standardized, reproducible, and clinically meaningful to influence patient management. In the pancreas, this issue is particularly complex because focal lesions are often heterogeneous, background parenchyma may be altered by fibrosis or obstructive pancreatitis, and stiffness may reflect multiple overlapping biological phenomena. In liver disease, the role of EUS-guided elastography must be interpreted against established non-invasive tools such as vibration-controlled transient elastography (VCTE), transabdominal 2D-SWE, serum-based fibrosis scores, and magnetic resonance elastography. EUS-guided elastography should therefore be regarded as an endoscopic, minimally invasive adjunct rather than a non-invasive screening tool. Its readiness for clinical practice should be considered separately for each acquisition mode and clinical indication rather than as a single global judgment; in particular, evidence from EUS-SWM or point SWE should not be automatically extrapolated to true EUS-guided 2D-SWE without dedicated validation.

This review critically appraises the physical principles, technical implementation, quality-control requirements, and current evidence regarding EUS-guided shear-wave-based elastography, including EUS-guided shear-wave measurement, point shear-wave elastography, and true two-dimensional shear-wave elastography. The main objective is to define where the technique appears clinically promising, where evidence remains insufficient, and what methodological steps are required before routine clinical adoption. In this review, clinical readiness is assessed according to technical feasibility, reliability of valid measurements, intra- and inter-observer reproducibility, inter-platform comparability, validated disease-specific thresholds, external validation, incremental value over established diagnostic pathways, workflow feasibility, procedural burden, safety, and evidence of impact on patient management.

Accordingly, this review primarily evaluates the current evidence for EUS-guided shear-wave-based elastography as a technological family. Evidence specifically supporting true real-time EUS-guided 2D-SWE is presented separately and should be considered preliminary.

## 2. Materials and Methods

This manuscript was developed as a narrative review. A literature search was performed in PubMed from database inception to May the Fourth 2026. using the following search string: ((“endoscopic ultrasound”[Title/Abstract] OR “endoscopic ultrasonography”[Title/Abstract] OR endosonography[Title/Abstract] OR EUS[Title/Abstract]) AND (“shear wave elastography”[Title/Abstract] OR “shear-wave elastography”[Title/Abstract] OR “shear wave measurement”[Title/Abstract] OR “shear-wave measurement”[Title/Abstract] OR SWE[Title/Abstract] OR SWM[Title/Abstract] OR “point shear wave elastography”[Title/Abstract] OR pSWE[Title/Abstract] OR “2D-SWE”[Title/Abstract] OR “two-dimensional shear wave elastography”[Title/Abstract] OR “two-dimensional shear-wave elastography”[Title/Abstract]) AND (pancreas[Title/Abstract] OR pancreatic[Title/Abstract] OR “pancreatic cancer”[Title/Abstract] OR “solid pancreatic lesion”[Title/Abstract] OR “chronic pancreatitis”[Title/Abstract] OR “early chronic pancreatitis”[Title/Abstract] OR “autoimmune pancreatitis”[Title/Abstract] OR liver[Title/Abstract] OR hepatic[Title/Abstract] OR “liver fibrosis”[Title/Abstract] OR MASLD[Title/Abstract] OR NAFLD[Title/Abstract] OR “metabolic dysfunction-associated steatotic liver disease”[Title/Abstract] OR “portal hypertension”[Title/Abstract] OR “endo-hepatology”[Title/Abstract] OR endohepatology[Title/Abstract] OR spleen[Title/Abstract] OR splenic[Title/Abstract])).

Original clinical studies, feasibility studies, reproducibility studies, comparative diagnostic studies, prospective and retrospective cohorts, and technical studies were statetment. Studies were included when they evaluated EUS-guided shear-wave-based elastography, EUS-guided shear-wave measurement, point shear-wave elastography, or true EUS-guided 2D-SWE in pancreatic, hepatobiliary, splenic, or endo-hepatology applications. Studies of transabdominal elastography were considered only when they provided relevant context or comparator data for liver stiffness assessment. Case reports, editorials without original data, duplicate reports, and studies not relevant to EUS-based shear-wave techniques were excluded from the main evidence synthesis. Only articles available in English or with sufficient English-language data for interpretation were included.

### 2.1. Terminology

For clarity, the term EUS-guided shear-wave-based elastography is used throughout this manuscript as an umbrella expression for EUS-based shear-wave techniques, including EUS-guided shear-wave measurement, point shear-wave elastography, and true two-dimensional shear-wave elastography. The specific acquisition mode is reported whenever available. Strictly speaking, 2D-SWE refers to techniques that generate a real-time two-dimensional elastographic map over a field of view, allowing quantitative stiffness assessment within one or more regions of interest. In contrast, EUS-SWM and point SWE provide quantitative measurements within a predefined sampling box or region, without necessarily displaying a full two-dimensional stiffness map. This distinction is essential because much of the available EUS literature uses EUS-SWM or point SWE rather than true 2D-SWE. Therefore, conclusions from EUS-SWM and point SWE studies are interpreted as evidence for EUS-guided shear-wave-based elastography in general, whereas claims specific to true EUS-guided 2D-SWE are restricted to studies using two-dimensional stiffness mapping.

Stiffness values may be expressed as shear-wave velocity, usually in meters per second (m/s), or as an elastic modulus, usually in kilopascals (kPa). Conversion between velocity and modulus assumes simplified tissue behavior and is influenced by tissue density, incompressibility, and viscoelasticity. Since biological tissues are not ideal elastic solids, values obtained from different systems, probes, acquisition modes, and processing algorithms should not be considered interchangeable without validation. When available, reliability metrics such as interquartile range-to-median ratio, confidence maps, net effective shear-wave velocity, valid acquisition rate, or system-specific quality indicators should be reported together with the raw stiffness measurements [2,5,6,7].

### 2.2. Statistics

Because the available evidence is heterogeneous in terms of device platform, acquisition mode, acquisition protocol, disease setting, reference standard, and outcome definition, the manuscript was structured as a narrative review rather than as a systematic review or meta-analysis. No formal pooled analysis was performed. The evidence tables summarize the principal clinical and technical studies identified for pancreatic and endo-hepatology applications, with emphasis on acquisition mode, reference standard, diagnostic performance, reproducibility, and major limitations.

### 2.3. AI Use Statement

A large language model (OpenAI (2026), ChatGPT (Versione GPT-4o, https://chatgpt.com)) was used to assist in the creation and graphical refinement of the graphical abstract. Artificial intelligence was not used for literature selection, study eligibility assessment, data extraction, interpretation of evidence, statistical analysis, or formulation of scientific conclusions. All AI-assisted material was critically reviewed and edited by the authors, who take full responsibility for the content of the manuscript.

## 3. Principles of Elastography

Elasticity refers to the ability of a material to return to its original shape after the removal of an external force. The amount of deformation induced by stress is termed strain and can be expressed as a relative change in length, area, or volume. Biological tissues exhibit complex mechanical behavior and cannot be described as purely elastic materials. Instead, they are typically non-linear, anisotropic, and viscoelastic. In viscoelastic materials, both elastic and viscous components contribute to mechanical response, meaning that tissue deformation depends not only on the applied force but also on time, microstructure, temperature, hydration, and tissue composition.

The measurement of tissue elasticity was first explored through mechanical vibration and tension experiments, which demonstrated that biological tissues cannot be adequately described as simple Hookean materials. These early mechanical observations established the conceptual link between tissue biomechanics and ultrasonography. Over time, advances in ultrasound technology, signal processing, and computational imaging led to the development of elastographic techniques capable of interrogating tissue stiffness in vivo.

In clinical ultrasound, elastographic techniques can be broadly divided into strain elastography and shear-wave-based elastography. Strain elastography evaluates tissue deformation induced by internal or external compression and provides qualitative or semi-quantitative information, usually displayed as a color-coded map, strain ratio, or histogram-derived parameter. It is therefore intrinsically comparative and depends on adequate compression, stable positioning, and selection of appropriate reference tissue [2,3,4]. Shear-wave-based elastography differs because it estimates stiffness by measuring the propagation velocity of mechanically induced shear-waves. In simplified elastic models, faster shear-wave propagation corresponds to higher stiffness, and measurements can be expressed as shear-wave velocity in m/s or as a derived elastic modulus in kPa. This quantitative output is attractive for diffuse parenchymal diseases such as chronic pancreatitis or liver fibrosis, where absolute or semi-absolute stiffness measurements may have clinical meaning. However, quantitative output should not be equated with automatic reproducibility or clinical validity. In vivo measurements remain vulnerable to motion, probe pressure, ROI placement, depth, tissue heterogeneity, and device-specific processing algorithms.

## 4. Physical and Technical Basis of Shear-Wave Elastography

Shear-wave elastography relies on the generation of localized tissue displacements by acoustic radiation force impulses (ARFI) or related push-pulse technologies. These impulses induce transverse shear-waves that propagate laterally through tissue. Ultrafast ultrasound imaging sequences then track shear-wave propagation and calculate shear-wave velocity. In an ideal incompressible elastic medium, the shear modulus (mu) is related to shear-wave velocity (cs) according to the equation mu = rho x cs squared, where rho represents tissue density. Under small-strain conditions, Young’s modulus can be approximated as E approximately equals 3 mu. Therefore, shear-wave velocity can be converted into quantitative stiffness values expressed either in m/s or kPa [2].

In vivo tissues, however, are viscoelastic and dispersive. Shear-wave speed may vary with frequency, tissue architecture, inflammation, fibrosis, fluid content, necrosis, and the direction of wave propagation. This is especially relevant in the pancreas, where lobular architecture, ductal structures, vascular pulsation, chronic inflammatory changes, and tumor heterogeneity may all influence the measured value. Consequently, SWE should be regarded as a quantitative surrogate of stiffness rather than a direct histological measurement [2,6].

Two main acquisition modes should be distinguished. Point SWE or shear-wave measurement estimates stiffness within a small predefined region of interest. Two-dimensional SWE generates a real-time color elastogram across a larger field of view and allows pixel-wise quantitative analysis. In EUS, this distinction is not always explicit in the literature, and many studies use the term EUS-SWE even when the system actually performs EUS-SWM. For manuscript interpretation and future study design, authors should report the specific equipment, software version, acquisition mode, region of interest, measurement depth, number of acquisitions, and quality criteria [5,6].

Many systems include reliability metrics such as interquartile range-to-median ratio, confidence maps, or net effective shear-wave velocity. These metrics are essential because technically successful image acquisition does not necessarily imply reliable stiffness measurement. The clinical value of EUS-guided SWE depends not only on mean stiffness values but also on the proportion of valid measurements, intra-observer reproducibility, inter-observer reproducibility, and inter-platform comparability [5,6,7,8].

### Acquisition Modes and Terminology: EUS-SWM, Point SWE, and 2D-SWE

Shear-wave-based elastography includes several acquisition modes that should not be used interchangeably. In the EUS literature, the terms shear-wave elastography, shear-wave measurement, point shear-wave elastography, and two-dimensional shear-wave elastography have sometimes been applied inconsistently, creating potential confusion when interpreting the available evidence [5,6]. Point shear-wave elastography and shear-wave measurement provide quantitative stiffness assessment within a predefined sampling box or small region of interest. The operator places the measurement box on the target tissue, and the system reports shear-wave velocity, usually expressed in meters per second, or a derived elastic modulus, usually expressed in kilopascals. This approach provides numerical stiffness data but does not necessarily generate a real-time two-dimensional stiffness map of the surrounding tissue. As a result, the validity of the measurement depends heavily on correct placement of the sampling box, avoidance of vessels, ducts, calcifications, cystic or necrotic components, and acquisition under stable technical conditions [5,6,7,8]. True two-dimensional shear-wave elastography differs from point-based methods because it generates a color-coded elastographic map over a wider field of view. Quantitative measurements can then be obtained from one or more regions of interest selected within this map. This technical feature may be clinically relevant because it allows the operator to visually identify more homogeneous and technically reliable areas for measurement while avoiding regions affected by artifacts, necrosis, vascular structures, ductal structures, or heterogeneous tumor components. In principle, this may improve the reliability of stiffness assessment in anatomically complex and heterogeneous organs such as the pancreas [5,6,7,8]. The distinction is particularly important for solid pancreatic lesions. Existing EUS-SWM and point SWE studies suggest that absolute stiffness values often overlap between pancreatic cancer, inflammatory masses, background pancreatic parenchyma, and normal tissue. This limitation may partly reflect biological heterogeneity, but it may also reflect technical constraints of point-based sampling. By allowing visual selection of the most appropriate measurement area, true 2D-SWE may potentially overcome some unresolved limitations of point SWE, including sampling error and inadvertent measurement of necrotic, cystic, vascular, or artifact-prone regions. However, this remains a hypothesis. At present, clinical evidence specifically validating true EUS-guided 2D-SWE for pancreatic cancer or other pancreatic indications is lacking, and conclusions from EUS-SWM or point SWE studies should not be directly extrapolated to 2D-SWE without dedicated validation [6,7,8].

Accordingly, this review uses “EUS-guided shear-wave-based elastography” as the umbrella term for EUS-SWM, point SWE/pSWE, and true 2D-SWE. When published clinical data are discussed, the specific acquisition mode is reported whenever available. True EUS-guided 2D-SWE should be regarded as an emerging technical evolution rather than an evidence-based replacement for currently published EUS-SWM and point SWE data. Diagnostic thresholds, reliability metrics, and clinical conclusions derived from one acquisition mode or device platform should not be transferred to another without dedicated validation.

## 5. Evolution of Elastography in Endoscopic Ultrasound

EUS elastography was initially developed as a strain-based technique. Early echoendoscopic systems lacked the technical capability to generate and track shear-waves in deep abdominal organs, and strain elastography therefore became the first clinically adopted approach. Strain elastography provides qualitative information through color mapping and semi-quantitative parameters such as strain ratios or histogram analysis. Early clinical studies demonstrated its potential value in the evaluation of pancreatic masses and lymph nodes, where malignant lesions typically exhibit reduced strain due to increased stiffness compared with surrounding tissue.

Despite these advantages, strain elastography remained inherently comparative and operator-dependent. Image quality depends on adequate compression, stable scope position, and selection of a suitable reference area. The resulting output is often useful as a visual adjunct but less suitable as a standardized biomarker. This limitation is particularly important when the clinical objective is to stage diffuse parenchymal disease, detect early fibrosis, or monitor longitudinal change.

Advances in ultrasound hardware, transducer bandwidth, frame rates, acoustic radiation force technology, and computational capacity have enabled the implementation of shear-wave-based techniques in EUS platforms. This technological development now allows quantitative measurement of stiffness in deep abdominal organs, including the pancreas, liver, and spleen. The shift from qualitative color patterns to quantitative measurements represents a major conceptual advance. However, quantitative output alone does not guarantee clinical readiness. For EUS-guided 2D-SWE to become a routine clinical tool, it must demonstrate reliable acquisition, reproducibility, validated disease-specific thresholds, and incremental value over conventional EUS, cross-sectional imaging, EUS-guided tissue acquisition, and established non-invasive tests [5,6,7].

## 6. Technical Considerations and Quality Control

Accurate EUS-guided shear-wave-based elastography requires standardized acquisition and reporting. Several technical factors may affect shear-wave propagation and measurement reliability, including respiratory motion, cardiac and vascular pulsation, peristalsis, patient movement, unstable contact between the echoendoscope and gastrointestinal wall, excessive probe pressure, measurement depth, small ROI size, and tissue heterogeneity. These issues are particularly relevant in the pancreas, where the target organ is small, anatomically curved, and surrounded by vascular and ductal structures. Scope position and acquisition route should be selected according to the target organ and clinical question. Pancreatic body and tail measurements are generally obtained from the stomach, whereas pancreatic head and uncinate measurements are usually obtained from the duodenal bulb or second duodenum. Liver measurements may be obtained from the stomach for the left lobe and from the duodenum for the right lobe, depending on local expertise and device availability. Each route has different susceptibility to cardiac motion, respiratory excursion, scope instability, and transducer pressure. Therefore, reports should specify the anatomical site, acquisition route, and scope position. Sedation and respiratory control may influence measurement stability. Deep sedation or general anesthesia may reduce voluntary motion but does not eliminate respiratory or cardiac artifacts. When feasible, measurements should be obtained during a stable respiratory phase, avoiding excessive torque, suction-related wall collapse, and excessive pressure from the echoendoscope tip. Probe pressure is particularly important because excessive compression may artificially increase measured stiffness, whereas unstable contact may lead to invalid propagation maps. Region of interest (ROI) placement should be standardized and tailored to the clinical indication. In chronic pancreatitis, many studies have focused on the pancreatic body because it is accessible and may allow more reproducible alignment; however, fibrosis may be heterogeneous across the gland, and body measurements may not represent disease involving the head, uncinate process, or tail. In focal pancreatic lesions, the ROI should avoid necrotic, cystic, vascular, calcified, or artifact-prone regions when possible. In liver applications, lobe selection should be reported because left-lobe measurements may be more influenced by cardiac motion, whereas right-lobe measurements may be technically more challenging depending on access route.

Quality control should distinguish technical acquisition from valid elastometry. A technically obtained image does not necessarily represent a reliable stiffness measurement. Future studies should report the number of attempted measurements, number of valid measurements, valid acquisition rate, stiffness distribution, median and interquartile range or mean and standard deviation, interquartile range (IQR)-to-median ratio when available, confidence maps, propagation maps, net effective shear-wave velocity or other system-specific quality metrics, and predefined criteria for excluding unreliable measurements. Failed measurements should be reported rather than omitted, because failure rate is clinically relevant for workflow feasibility. Workflow integration is also important. In pancreatic disease, EUS-guided elastography should be performed without delaying clinically indicated tissue acquisition or therapeutic intervention. In endo-hepatology, EUS-SWE may be most efficient when combined with procedures already requiring EUS, such as excluding other causes of abnormal liver test results (e.g., biliary stone disease), performing EUS-guided liver biopsy, or measuring the portal pressure gradient. In contrast, using EUS-SWE solely as a first-line fibrosis assessment would usually be difficult to justify in terms of costs and invasiveness when VCTE, transabdominal SWE, serum biomarkers, or magnetic resonance elastography are available.

A minimum reporting set should be adopted in future studies and in clinical protocols. Investigators should report the EUS platform and software, acquisition mode, stiffness unit, anatomical site, patient position and sedation strategy, scope position, ROI size, measurement depth, number of attempted and valid acquisitions, quality metrics, exclusion criteria for invalid measurements, and whether values are reported as median with interquartile range or mean with standard deviation. Without these details, comparison between studies and translation into clinical practice remain problematic.

The need for standardization has been emphasized by technical and feasibility studies. Prospective pancreatic data suggest that EUS-guided shear-wave-based elastography can be safe, feasible, and reproducible under controlled conditions, but reliability varies by pancreatic region. Technical studies also show that depth, ROI size, tissue heterogeneity, and acquisition conditions substantially influence measurements (summarized in Table 1). Therefore, standardization should not be considered a methodological detail but a prerequisite for clinical implementation [5,6,7,8].

Reported stiffness values and diagnostic thresholds should be considered platform-specific. Differences in acoustic radiation force generation, tracking pulse sequences, transducer geometry, echoendoscope design, software version, signal-processing algorithms, ROI dimensions, depth, and quality-control parameters may produce systematically different measurements. Consequently, a threshold derived using one vendor or acquisition mode should not be applied to another platform without direct cross-platform calibration and external validation. Similarly, values expressed in m/s and kPa should not be regarded as automatically interchangeable, because conversion to an estimated elastic modulus assumes a homogeneous, isotropic, incompressible, and purely elastic medium, assumptions that are not fully met by pancreatic or hepatic tissue.

## 7. Clinical Applications

The clinical role of EUS-guided 2D-SWE and related shear-wave techniques differs substantially across indications. Evidence is most coherent when stiffness reflects a diffuse or relatively homogeneous parenchymal process, such as chronic pancreatitis, autoimmune pancreatitis, or liver fibrosis. Evidence is less convincing when SWE is applied to focal pancreatic lesions, where stiffness may be influenced by necrosis, desmoplasia, inflammation, background fibrosis, and tumor heterogeneity. For this reason, the following sections are organized according to clinical setting and degree of evidence rather than technology alone.

The interpretation of EUS-guided shear-wave-based elastography in chronic pancreatitis should also account for disease heterogeneity. Alcohol-related CP, obstructive CP, autoimmune pancreatitis evolving toward chronicity, hereditary pancreatitis, and metabolic or fatty pancreas-related disease may exhibit different distributions of fibrosis, inflammation, fatty replacement, calcification, and ductal remodeling. These differences may produce distinct stiffness profiles even among patients with similar clinical severity. Therefore, a single universal cut-off is unlikely to be appropriate across all CP subtypes. Future studies should evaluate whether stiffness thresholds require stratification by etiology, disease stage, pancreatic region, and acquisition protocol. An additional methodological limitation is the potential for incorporation bias. Several CP studies use JPSC criteria, Rosemont features, conventional EUS findings, or composite criteria as the reference standard. Because these criteria include pancreatic parenchymal or ductal abnormalities that may also influence stiffness, diagnostic performance estimates should be interpreted with caution. EUS-guided shear-wave-based elastography should therefore be considered an adjunct to established criteria rather than an independent replacement until externally validated against robust clinical, functional, and longitudinal outcomes.

### 7.1. Chronic Pancreatitis and Early Chronic Pancreatitis

**Figure 1:**
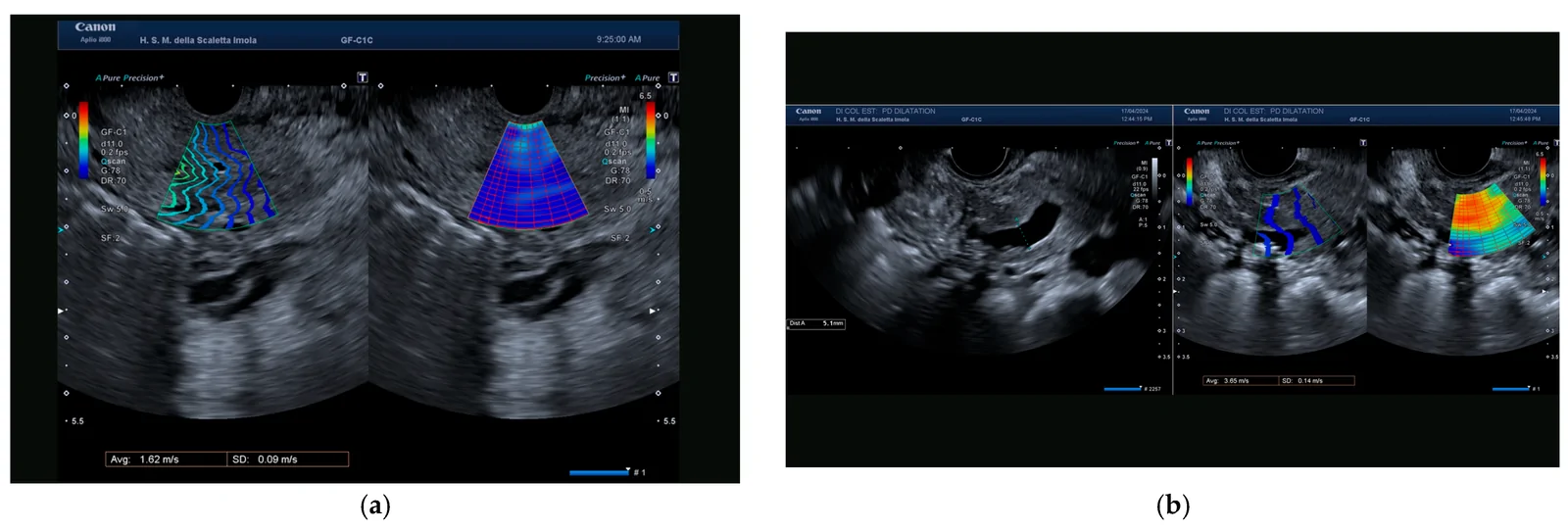
EUS-guided shear-wave-based elastography of pancreatic parenchyma: EUS platform Canon Aplio i800 (Tokyo, Japan) coupled with Olympus UCT-180 echoendoscope (Tokyo, Japan); ROI diameter, 1 cm. (**a**) Normal pancreatic parenchyma showing low shear-wave velocity/stiffness values (1.62 ± 0.09 m/s). (**b**) Chronic pancreatitis with main pancreatic duct dilation and increased shear-wave velocity/stiffness values (3.65 ± 0.14 m/s). The region of interest/measurement box is shown within the elastography field. Color scale interpretation: blue indicates lower stiffness and red/orange indicates higher stiffness according to the device-specific scale.

Chronic pancreatitis (CP) is characterized by progressive and irreversible fibrotic remodeling of the pancreatic parenchyma, accompanied by acinar cell loss, ductal distortion, inflammatory infiltration, and eventual calcification. This structural transformation leads to increased tissue rigidity, providing a strong biological rationale for the use of quantitative elastographic techniques as objective biomarkers of disease progression. Compared with focal pancreatic cancer, CP may represent a more suitable target for SWE because the clinical objective is often assessment of diffuse parenchymal remodeling rather than discrimination of one focal lesion from a heterogeneous background (Figure 1).

Multiple studies have reported promising diagnostic performance for EUS-SWE or EUS-SWM in differentiating CP from normal pancreatic tissue. Reported cut-off values vary across investigations, but diagnostic accuracy has generally been encouraging, with AUROC values frequently exceeding 0.80. Yamashita et al. reported that EUS-SWM increased with CP severity and could provide an objective assessment of pancreatic fibrosis. In a subsequent study, SWE also correlated with pancreatic exocrine and endocrine dysfunction, suggesting that stiffness may reflect not only morphological damage but also clinically relevant functional impairment [9,10,11,12,13,14].

Early chronic pancreatitis is an especially relevant potential indication. Conventional diagnostic criteria may be insensitive in early disease, and EUS features can be subtle, subjective, and prone to interobserver variability. Shintani et al. reported significantly higher shear-wave velocity in patients with early CP compared with matched controls, with an AUROC of 0.82 and a proposed cut-off of 2.24 m/s. These findings suggest that EUS-SWE may help quantify early fibrotic remodeling before overt advanced structural changes are present. However, early CP remains a challenging diagnostic entity, and future studies should determine whether increased stiffness predicts clinical progression, pain recurrence, exocrine insufficiency, endocrine dysfunction, or response to risk-factor modification [12,13].

The variability in reported thresholds likely reflects methodological heterogeneity. Differences in study populations and patient selection, such as early versus advanced CP, alcohol-related versus idiopathic disease, or coexistence of metabolic comorbidities, may influence baseline parenchymal stiffness. ROI placement is another critical determinant. Although measurements from the pancreatic body may facilitate consistent anatomical access, depth of measurement, insonation angle, respiratory motion, probe pressure, and exclusion of vascular or ductal structures have not been standardized across studies.

Etiology-specific differences may also alter patterns of fibrosis and stiffness distribution. Alcohol-related CP, autoimmune pancreatitis evolving toward chronicity, hereditary pancreatitis, obstructive pancreatitis, and metabolic pancreatic injury may exhibit distinct spatial patterns of fibrosis, calcification, inflammation, and fatty replacement. These differences may produce divergent shear-wave velocity ranges even among patients with comparable clinical severity. Therefore, CP-specific thresholds may need to be stratified by disease stage, etiology, anatomical region, and acquisition protocol. Among pancreatic applications, CP currently appears to be the most plausible indication for EUS-guided SWE. Nevertheless, the technique is not yet ready to replace established diagnostic criteria. Its role should be considered adjunctive: it may provide objective quantification of parenchymal stiffness, support diagnosis in equivocal cases, and potentially contribute to staging or functional risk stratification. Clinical adoption will require multicenter validation, standardized thresholds, and demonstration that SWE improves diagnostic confidence or management beyond conventional EUS criteria and pancreatic function tests. Clinical evidence on the use of EUS-SWE in chronic pancreatitis is summarized in Table 2 [7,8,9,10,11,12,13,14,15,16,17,18,19,20,21].

### 7.2. Autoimmune Pancreatitis

Autoimmune pancreatitis (AIP) is characterized by inflammatory and fibrotic remodeling of the pancreas, often with diffuse or segmental enlargement and increased tissue rigidity. From a pathophysiological standpoint, type 1 AIP is associated with lymphoplasmacytic infiltration, storiform fibrosis, and obliterative phlebitis. These features provide a biological rationale for increased shear-wave velocity during active disease. In contrast to pancreatic cancer, where intratumoral heterogeneity and adjacent obstructive changes may complicate stiffness interpretation, AIP may produce more diffuse and homogeneous stiffness changes, potentially favoring reproducible SWE assessment.

Preliminary clinical data support this rationale. In a prospective exploratory study, Ohno et al. evaluated EUS-SWM for assessment of AIP activity. Patients with AIP showed significantly higher pancreatic stiffness than subjects with normal pancreas. In the pancreatic body, median shear-wave velocity was 2.57 m/s in AIP compared with 1.89 m/s in controls. In a small subgroup of six patients reassessed after corticosteroid therapy, shear-wave velocity decreased significantly from 3.32 m/s to 2.46 m/s after short-term prednisone treatment. This rapid reduction likely reflects resolution of inflammatory infiltration and edema, with early modulation of tissue stiffness [15].

These findings suggest that EUS-SWE may be particularly useful as an adjunctive biomarker of inflammatory activity and treatment response rather than as a standalone diagnostic test for AIP. This distinction is important. The diagnosis of AIP relies on a composite assessment including imaging, serology, other organ involvement, histology, and response to steroids. SWE cannot replace this framework. However, quantitative stiffness assessment may add objective information when evaluating baseline activity, monitoring early response, or distinguishing persistent fibrotic remodeling from ongoing inflammation.

Several limitations should be emphasized. The available AIP data are based on small cohorts, and only a limited number of patients have undergone longitudinal post-treatment evaluation. Validated cut-offs for active disease, remission, relapse, or fibrotic sequelae are lacking. The relationship between stiffness values and serum IgG4, histological activity, imaging response, and long-term outcomes remains uncertain. Therefore, EUS-guided SWE in AIP should currently be regarded as a promising research and adjunctive monitoring tool rather than a routine diagnostic requirement.

### 7.3. Solid Pancreatic Lesions and Pancreatic Cancer

Evidence regarding the clinical role of EUS-SWE in solid pancreatic lesions, particularly pancreatic ductal adenocarcinoma (PDAC), is more limited and less consistent. From a biological perspective, PDAC is often characterized by a prominent desmoplastic reaction and abundant fibrotic stroma, which would be expected to increase stiffness. However, clinical elastographic findings have been inconsistent because measured stiffness reflects not only tumor fibrosis but also necrosis, cellularity, inflammatory infiltrates, vascularity, obstructive pancreatitis, and background parenchymal remodeling.

Although current evidence does not support EUS-guided shear-wave-based elastography as a standalone diagnostic test for solid pancreatic lesions, stiffness mapping may have potential adjunctive roles. In heterogeneous tumors, elastographic maps may help identify regions that are more suitable for tissue acquisition while avoiding necrotic, cystic, vascular, or artifact-prone areas. In principle, this could improve biopsy targeting, reduce false-negative EUS-guided tissue acquisition, or guide repeat biopsy after nondiagnostic EUS-FNA/FNB. These potential applications may be particularly relevant when stiffness information is interpreted together with B-mode morphology, Doppler, contrast-enhanced EUS, cross-sectional imaging, and clinical risk factors. However, this role remains hypothetical. No adequately powered prospective study has demonstrated that EUS-guided stiffness mapping improves diagnostic yield, reduces false-negative biopsy results, or changes patient outcomes in solid pancreatic lesions. Therefore, stiffness mapping should not delay or replace EUS-guided tissue acquisition. Its possible value should be investigated in prospective studies evaluating predefined biopsy-targeting strategies, diagnostic yield, number of passes, false-negative rate, and clinical impact.

Several studies have shown that absolute shear-wave velocity values may overlap between malignant lesions, surrounding pancreatic parenchyma, inflammatory masses, and normal pancreatic tissue. Kojima et al. evaluated patients with pancreatic cancer and individuals with normal pancreas and observed mean shear-wave velocity values that did not differ substantially among cancer lesions, adjacent background parenchyma, and normal pancreatic tissue. This overlap substantially limits the diagnostic capability of EUS-SWE when absolute stiffness values are used alone [16,17].

Relative stiffness parameters may be more informative. In the same study, normalized shear-wave velocity, which expresses lesion stiffness relative to surrounding parenchyma, showed better discriminatory performance than absolute velocity values. VsN values were lower in pancreatic cancer lesions compared with background pancreatic parenchyma and normal pancreas, and a VsN threshold of 50% achieved high AUROC for differentiating malignant from non-malignant tissue. These results suggest that relative or normalized parameters may partially compensate for interindividual variability in baseline parenchymal stiffness. However, such parameters require external validation before clinical use.

In a retrospective comparative study of solid pancreatic lesions, Ohno et al. compared EUS-SWM with strain elastography and strain-hue histogram analysis. Mean shear-wave velocity values differed numerically among pancreatic cancer, pancreatic neuroendocrine neoplasms, and mass-forming pancreatitis, but these differences did not reach statistical significance. In contrast, strain-based parameters demonstrated greater discriminatory capacity between pancreatic cancer and inflammatory lesions. These findings indicate that the quantitative nature of SWE does not automatically translate into superior diagnostic performance for focal lesions [16,18].

The limited performance of SWE in pancreatic cancer likely reflects the marked biological heterogeneity of pancreatic tumors and surrounding tissue. Regions of dense desmoplastic stroma may be stiff, whereas necrotic or cystic areas may be softer. Tumors causing main pancreatic duct obstruction may induce upstream inflammatory and fibrotic changes, and increased background stiffness and reducing lesion-to-parenchyma contrast. Conversely, pancreatic cancer arising in chronic pancreatitis may be difficult to distinguish because both the lesion and the surrounding parenchyma may be stiff. Small lesion size further limits the placement of homogeneous regions of interest and increases measurement error. At present, EUS-guided 2D-SWE or SWM should not be considered a replacement for conventional EUS morphology, contrast-enhanced EUS when available, cross-sectional imaging, or EUS-guided tissue acquisition in the diagnostic work-up of solid pancreatic lesions. Its role may be adjunctive, potentially supporting risk stratification or biopsy targeting in selected cases, but current evidence does not support its use as a standalone diagnostic tool for pancreatic cancer.

### 7.4. Endo-Hepatology

Transabdominal 2D-SWE has emerged as a robust and clinically valuable modality for the non-invasive assessment of liver fibrosis in patients with chronic liver disease. By providing quantitative measurements of liver stiffness expressed in kPa or m/s, 2D-SWE allows clinicians to stratify fibrosis stage, identify advanced disease, and monitor longitudinal changes. In comparative studies, transabdominal 2D-SWE and VCTE have shown comparable diagnostic performance for advanced fibrosis and cirrhosis, and 2D-SWE has been evaluated using cut-offs derived from established non-invasive fibrosis frameworks [21].

The potential role of EUS-guided shear-wave-based elastography in endo-hepatology should be balanced against procedural burden. Unlike VCTE, serum biomarkers, magnetic resonance elastography, or transabdominal 2D-SWE, EUS-guided elastography requires endoscopy, sedation, endoscopy-room resources, trained personnel, and procedure time. It also carries the general risks and costs associated with upper gastrointestinal endoscopy, even when no tissue acquisition is performed. For this reason, EUS-SWE should not be considered a first-line replacement for established non-invasive fibrosis tests in unselected patients. Its most appropriate niche may be in selected patients already undergoing EUS for another indication, patients in whom transabdominal techniques are unreliable or fail, and patients requiring a one-step endo-hepatology evaluation combining liver stiffness assessment, EUS-guided liver biopsy, and portal pressure gradient measurement. In these settings, the incremental value of EUS-SWE should be judged not only by diagnostic accuracy, but also by whether it reduces diagnostic uncertainty, avoids additional procedures, improves risk stratification, or changes clinical management.

EUS-guided SWE has recently been extended to the endo-hepatology setting. In this context, its value may derive less from replacing established transabdominal techniques and more from integration into a comprehensive EUS-based diagnostic session. EUS can provide liver stiffness measurement, EUS-guided liver biopsy, and direct portal pressure gradient assessment in the same procedure. This combined approach may be particularly attractive in patients requiring endoscopic evaluation, patients with obesity, patients with indeterminate non-invasive tests, or patients in whom simultaneous histological and hemodynamic assessment is clinically relevant [22,23,24,25,26].

Comparative studies have shown that EUS-SWE may perform similarly to VCTE for fibrosis staging and may correlate with histological fibrosis. Kohli et al. reported that endosonographic SWE correlated with liver histology and had accuracy comparable to VCTE. Importantly, VCTE may be unreliable or fail in some patients in whom EUS-SWE remains technically feasible. These findings support the concept that EUS-SWE can provide clinically meaningful liver stiffness measurements, although standardization of lobe selection, acquisition route, and quality criteria remains necessary [22].

Technical nuances are particularly important in liver applications. Reproducibility studies have reported that paired consecutive EUS-SWE measurements may vary by hepatic lobe, with greater variance in the left lobe than the right lobe. This discrepancy probably reflects differences in acoustic window, cardiac motion, respiratory excursion, and transducer contact stability. Therefore, liver EUS-SWE protocols should specify whether measurements are obtained from the left lobe, right lobe, or both; the acquisition route; the number of measurements; and the reliability criteria used to define valid results [23].

One of the most clinically relevant potential advantages of EUS-SWE pertains to patients with obesity and metabolic dysfunction-associated steatotic liver disease. In individuals with elevated body mass index, transabdominal VCTE may yield unreliable or failed measurements due to subcutaneous adipose tissue thickness and ultrasound attenuation. In a multicenter cross-sectional pilot study of patients with obesity and MASLD, EUS-SWE showed superior accuracy compared with FIB-4 and VCTE for fibrosis staging. The enhanced performance of EUS-SWE in this population likely derives from the close proximity of the EUS transducer to the liver parenchyma through the gastric or duodenal wall, thereby minimizing the influence of subcutaneous adiposity [24].

Beyond fibrosis staging, shear-wave techniques have been explored for assessment of portal hypertension through splenic stiffness measurement. Sustained elevation of portal venous pressure leads to splenic congestion, sinusoidal dilatation, reticuloendothelial hyperplasia, and fibrogenic remodeling, all of which can increase splenic stiffness. Although most splenic elastography evidence is transabdominal, the physiological rationale is relevant to EUS-based endo-hepatology. Future EUS studies should clarify whether liver and splenic stiffness, alone or combined with EUS-guided portal pressure gradient measurement, can improve non-invasive risk stratification for clinically significant portal hypertension and varices [27].

The most sophisticated application of EUS-SWE may be as part of a one-step endo-hepatology platform. Studies combining EUS-guided liver elastography, EUS-guided portal pressure gradient measurement, and EUS-guided liver biopsy have demonstrated high technical success and correlation between stiffness, portal pressure, and histological fibrosis stage. This integrated approach is conceptually attractive because it links structural, functional, and histological assessment. Nevertheless, it should be interpreted as an emerging specialized application rather than a broadly established standard of care [26].

**Figure 2:**
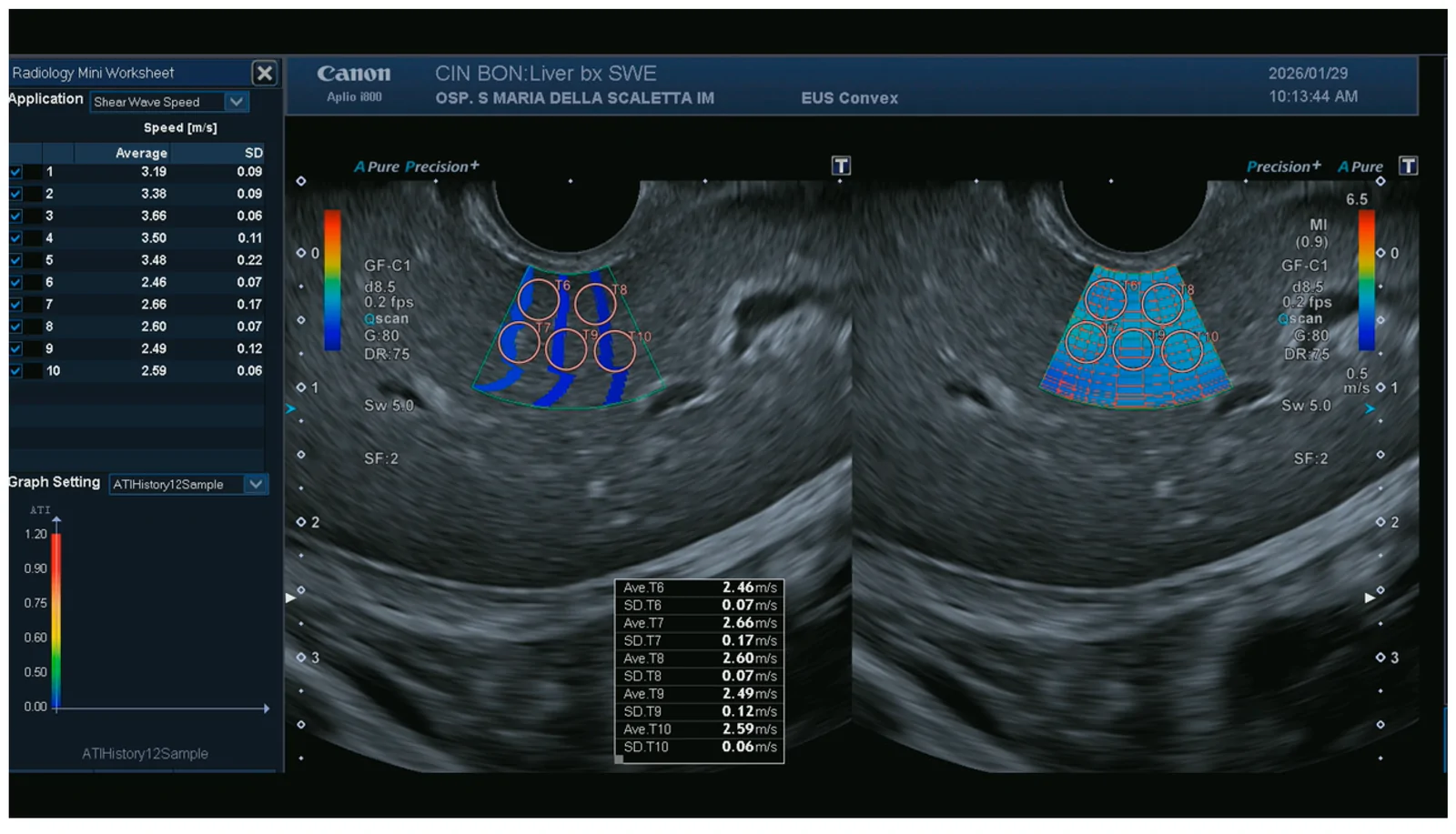
2D-SWE of left liver lobe in a patient with elevated liver function test, showing increased velocity, despite normal B-mode appearance of liver parenchyma, suggesting underlying fibrosis.

Figure 2 EUS-guided shear-wave-based elastography (EUS platform Canon Aplio i800, Tokyo, Japan, coupled with Olympus UCT-180 linear-array EUS echoendoscope, Tokyo, Japan; ROI diameter, 1 cm, with the ROI placed at a depth of 1–2 cm) of the left liver lobe in a patient undergoing endo-hepatology assessment for elevated liver function tests and ruling-out biliary stone disease. The elastography field and measurement ROI regions (round) are shown within the liver parenchyma. Increased shear-wave velocity/stiffness values indicate increased liver stiffness according to the device-specific color scale; however, fibrosis staging requires interpretation in combination with an accepted reference standard such as histology, validated non-invasive tests, imaging, clinical context, or portal pressure assessment. The obtained 10 stiffness measurements are shown in the upper left box of the images; for each measurement, velocity (m/s) and standard deviation (SD) are reported.

Clinical applications of EUS-guided shear-wave-based elastography in endo-hepatology have been summarized in Table 3.

## 8. Readiness for Clinical Practice

The readiness of EUS-guided 2D-SWE for clinical practice should be judged according to indication, not technology alone. A technique may be technically feasible but clinically immature if it lacks standardized protocols, validated thresholds, reproducibility across operators and systems, and evidence of incremental value. From this perspective, current evidence supports four different levels of readiness.

First, in chronic pancreatitis, EUS-guided SWE appears promising and may soon become a useful adjunct for objective assessment of pancreatic stiffness. Evidence suggests correlation with CP severity, early CP criteria, and pancreatic functional impairment. However, before routine use, thresholds need to be validated across centers, systems, and disease etiologies, and studies must demonstrate clinical impact beyond conventional EUS criteria.

Second, in autoimmune pancreatitis, SWE may be useful as a quantitative marker of disease activity and steroid response. Current evidence is biologically coherent but based on small cohorts. Its most plausible role is longitudinal monitoring rather than primary diagnosis.

Third, in solid pancreatic lesions, particularly pancreatic cancer, EUS-guided shear-wave-based elastography is not ready for routine diagnostic use as a standalone tool. Absolute stiffness values overlap between malignant and benign or inflammatory conditions, and relative metrics require validation. EUS-guided tissue acquisition remains central to diagnosis.

Fourth, in endo-hepatology, EUS-guided shear-wave-based elastography is promising in selected populations, especially patients with obesity, indeterminate non-invasive fibrosis assessment, or a need for combined liver biopsy and portal pressure measurement. Its broader use will depend on whether it offers added value over VCTE, transabdominal 2D-SWE, MR elastography, and serum-based fibrosis scores.

## 9. Critical Appraisal of the Current Evidence Base

The current evidence base for EUS-guided shear-wave-based elastography should be interpreted cautiously. Most studies are exploratory, single-center investigations involving relatively small and selected populations. Retrospective designs, heterogeneous acquisition protocols, inconsistent definitions of measurement validity, and limited reporting of failed acquisitions reduce comparability across studies. External validation is uncommon, and disease-specific thresholds have generally been derived and evaluated within the same cohort. An additional concern is the choice of reference standard. In chronic and early chronic pancreatitis, several studies used Rosemont criteria, Japan Pancreas Society criteria, or composite EUS-based definitions. Because these classifications include parenchymal and ductal abnormalities that may themselves be associated with increased stiffness, incorporation bias may have contributed to apparently high diagnostic accuracy. In solid pancreatic lesions, histological confirmation has not always been available in all participants, and the biological overlap among desmoplasia, inflammation, necrosis, fibrosis, and obstructive pancreatitis limits the specificity of absolute stiffness measurements. Technical feasibility should also be distinguished from clinical utility. High technical success and reproducibility under controlled conditions do not establish that the technique changes diagnosis, avoids tissue acquisition, improves risk stratification, guides treatment, or affects patient outcomes. At present, no sufficiently validated evidence demonstrates that EUS-guided shear-wave elastography improves management beyond established diagnostic pathways. Therefore, the available data support further prospective validation rather than routine standalone use.

## 10. Incremental Clinical Value Relative to Established Diagnostic Modalities

The potential value of EUS-guided shear-wave-based elastography should be defined in relation to existing diagnostic pathways rather than on the basis of diagnostic accuracy alone. In pancreatic disease, conventional EUS, cross-sectional imaging, contrast-enhanced EUS, strain elastography, pancreatic function testing, and EUS-guided tissue acquisition already provide complementary morphological, vascular, functional, and histological information. Shear-wave measurement may add objective quantitative information, particularly in diffuse pancreatic disease or longitudinal assessment, but it cannot currently replace tissue acquisition when pathological confirmation is required. In liver disease, VCTE, transabdominal SWE, serum-based fibrosis scores, and magnetic resonance elastography are non-invasive or less invasive first-line options. EUS-guided SWE therefore appears difficult to justify as an isolated screening test. Its potential incremental value is more plausible in patients already undergoing EUS, in individuals with technically unreliable transabdominal measurements, or as part of a comprehensive endo-hepatology procedure combining stiffness assessment with EUS-guided liver biopsy and portal pressure measurement. Future studies should evaluate whether the addition of EUS-guided SWE results in reclassification, fewer biopsies, improved diagnostic confidence, altered management, or better clinical outcomes (Table S1).

## 11. Limitations and Future Directions

Despite promising developments, several limitations currently restrict widespread clinical adoption of EUS-guided 2D-SWE. Technical limitations include motion artifacts, cardiac and vascular pulsation, unstable transducer contact, excessive probe pressure, small or heterogeneous regions of interest, depth-related signal attenuation, and system-specific processing algorithms. These factors are particularly relevant in the pancreas, where the target organ is small, deep, anatomically curved, and frequently heterogeneous.

Biological limitations also deserve emphasis. Pancreatic stiffness may reflect fibrosis, inflammation, edema, fatty replacement, necrosis, desmoplasia, ductal obstruction, or tumor cellularity. These processes may coexist within the same patient and even within the same lesion. Therefore, a single stiffness value may not map directly onto a single pathological diagnosis. In liver disease, stiffness may be influenced by fibrosis, inflammation, cholestasis, congestion, steatosis, and portal pressure, and interpretation should always consider clinical context [27,28,29].

Methodological limitations of the current evidence include small sample size, single-center design, heterogeneous inclusion criteria, inconsistent acquisition protocols, variable reference standards, limited interobserver data, and lack of external validation. Many studies are exploratory and report diagnostic accuracy against imperfect or composite reference standards. Few studies evaluate whether EUS-guided shear-wave-based elastography changes clinical decision-making, improves patient outcomes, reduces the need for additional testing, or provides cost-effective information. Future research should be multicenter, disease-specific, and protocolized. Studies should prespecify acquisition parameters, quality criteria, anatomical site, number of measurements, and statistical handling of failed or unreliable acquisitions. Disease-specific cut-offs should be validated externally and should not be transferred uncritically across systems or anatomical regions. In chronic pancreatitis, future endpoints should include progression, pain, exocrine insufficiency, endocrine dysfunction, and response to treatment. In AIP, longitudinal studies should correlate stiffness with clinical activity, IgG4 status, imaging response, histology, relapse, and steroid responsiveness. In pancreatic cancer, studies should evaluate whether normalized stiffness parameters, elastographic heterogeneity, or combined multiparametric EUS models can improve diagnosis or biopsy targeting. In liver disease, EUS-guided shear-wave-based elastography should be compared with VCTE, transabdominal 2D-SWE, MR elastography, serum biomarkers, histology, and portal pressure measurements. A key priority for future research is the standardization of terminology and reporting in EUS-guided shear-wave-based elastography [3]. Current studies variably use the terms EUS-SWE, EUS-SWM, point SWE, and 2D-SWE, sometimes interchangeably, despite important technical differences between these acquisition modes. This inconsistency limits comparison across studies and may lead to inappropriate extrapolation of diagnostic thresholds from one technology or platform to another [29,30,31,32]. Future publications should clearly specify the acquisition technique used, distinguishing true two-dimensional SWE from point SWE or shear-wave measurement, and should report device manufacturer, software version, probe type, anatomical site, ROI size and depth, number of attempted and valid measurements, quality metrics, and statistical handling of unreliable acquisitions. A minimum reporting framework is proposed in Table 1, with the aim of improving reproducibility, facilitating multicenter validation, and supporting the development of disease-specific thresholds for clinical implementation.

Device dependence remains a central limitation. EUS-guided shear-wave-based elastography measurements are influenced by manufacturer, echoendoscope, transducer characteristics, software version, acquisition mode, ROI size, depth, signal-processing algorithm, and quality-control metrics. Consequently, stiffness values, cut-offs, and reproducibility estimates should be considered platform-specific until validated across systems. Inter-platform comparability cannot be assumed, and future multicenter studies should either standardize equipment or include device-specific analyses. This issue is particularly relevant when interpreting studies conducted with different vendors or when disclosed industry relationships involve elastography-capable platforms.

Finally, reporting standards are urgently needed. A consensus checklist for EUS-guided SWE studies should include terminology, device information, acquisition mode, ROI characteristics, measurement depth, anatomical site, quality metrics, number of valid measurements, reproducibility analysis, and reference standard (Table 1). Without such standardization, pooled analyses and clinical translation will remain limited.

## 12. Conclusions

EUS-guided shear-wave-based elastography is technically feasible and provides quantitative information on tissue mechanical properties. However, clinical readiness differs according to acquisition mode and indication. The most consistent evidence currently concerns EUS-SWM and point SWE in chronic pancreatitis, early chronic pancreatitis, autoimmune pancreatitis, and selected endo-hepatology settings. Direct evidence supporting true EUS-guided 2D-SWE remains limited.

Current studies are affected by small sample sizes, heterogeneous protocols, device-specific quality metrics, limited external validation, possible incorporation bias, and insufficient evidence of incremental clinical value. Absolute stiffness values and proposed cut-offs should not be transferred across vendors, software versions, acquisition modes, or anatomical sites without dedicated validation [9,10,11,12,13,14,15].

EUS-guided shear-wave elastography should therefore be considered an adjunctive component of multiparametric EUS rather than a standalone diagnostic test. Its most plausible future role lies in quantitative assessment of diffuse pancreatic disease, longitudinal monitoring, and integrated endo-hepatology procedures in selected patients already undergoing EUS. Prospective multicenter studies should prioritize standardized acquisition, cross-platform reproducibility, independent reference standards, external validation, and demonstration of a measurable impact on clinical decision-making.

Overall, EUS-guided 2D-SWE is technically feasible and clinically promising, but not yet broadly ready for routine standalone use. Its future role will depend on multicenter validation, reproducibility across systems and operators, disease-specific thresholds, and demonstration that quantitative stiffness assessment improves clinical decision-making beyond existing diagnostic pathways.

## Figures and Tables

**Table 1:** Proposed minimum reporting items for EUS-guided shear-wave-based elastography studies.

Domain	Items to Report	Rationale
EUS platform	Manufacturer, echoendoscope, processor, software version, probe/transducer, acquisition mode	Stiffness values and quality metrics may not be interchangeable across systems
Patients and procedural details	Sedation, patient position, fasting status, respiratory control, procedural indication	Motion and stability influence reliability and workflow feasibility
Anatomy and shear-wave measurement route	Organ, pancreatic region or liver lobe, access route, scope position	Stiffness and reproducibility vary by anatomical site and acquisition route
Region of interest (ROI)	ROI size, depth, number of ROIs, placement criteria, avoidance of vessels, ducts, calcifications, cystic or necrotic areas	ROI selection is a major determinant of measurement validity
Shear-wave measurement acquisition	Number of attempted measurements, number of valid measurements, valid acquisition rate, failed measurements	Technical success does not equal valid elastometry.
Metrics	IQR/median, propagation map, confidence map/index, net effective shear-wave velocity, system-specific reliability indicator, normalized velocity if used.	Quality metrics are necessary to interpret unreliable or variable values.
Statistics	Median/IQR or mean/SD, handling of failed measurements, predefined cut-offs, AUROC, sensitivity, specificity, reproducibility statistics	Statistical handling affects comparability and diagnostic thresholds
Clinical context	Reference standard, intended clinical use, comparator test, timing between index test and reference standard	Defines whether the technique is diagnostic, prognostic, monitoring, or adjunctive
Limitations	Sample size, single-center design, retrospective design, missing reference standard, incorporation bias, device specificity, selection bias	Pivotal role to judge clinical applicability and readiness

**Table 2:** Pancreatic applications of EUS-guided shear-wave-based elastography.

Study, Reference	Design and Population	EUS Platform and Acquisition Mode	ROI, Acquisition Protocol and Quality Control	Reference Standard	Main Quantitative Findings	Feasibility and Reproducibility	Major Limitations and Clinical Interpretation
Abboud et al. 2023 [7]	Prospective cohort of consecutive patients undergoing clinically indicated pancreatic EUS; feasibility and reproducibility study rather than diagnostic accuracy study.	EUS-SWE/shear-wave-based pancreatic measurements; vendor and software version NR in the manuscript table and abstract-level source.	Ten shear-wave velocity readings in each pancreatic region: head, body, and tail. Reliability assessed using net effective shear-wave velocity percentage (VsN); VsN > 50% considered reliable.	Technical success, safety, valid acquisition rate, and reproducibility metrics; no disease-specific diagnostic reference standard.	117 patients; 3320 measurements. Technical success 100%. Reliable measurements: head 85.1%, body 75.5%, tail 64.2%. Reliability varied significantly by region.	No procedure-related complications reported. Intraclass correlation coefficient ranged from 0.80 to 0.89, supporting high reproducibility under controlled acquisition conditions.	Supports feasibility and need for regional quality control, but does not validate disease-specific cut-offs.
Yamashita et al. 2020 [9]	Prospective observational study; 52 patients undergoing pancreatic EUS, classified from normal/indeterminate findings to chronic pancreatitis.	EUS-SWM; point/box-based shear-wave measurement rather than true real-time 2D-SWE mapping. Vendor/software details NR in the original summary table.	Median shear-wave velocity used for analysis. ROI location, number of acquisitions, and valid acquisition criteria should be detailed in the final manuscript if available from full text.	Rosemont classification/EUS findings used to classify non-CP and CP groups.	AUROC for CP diagnosis 0.97. Proposed cut-off 2.19 m/s; sensitivity 100%, specificity 94%. For endocrine dysfunction in CP: AUROC 0.75; cut-off 2.78 m/s; sensitivity 70%, specificity 56%.	Specific valid acquisition rate and reproducibility metrics NR in the manuscript table.	Promising diagnostic performance, but the reference standard includes EUS parenchymal features potentially related to tissue stiffness, creating possible incorporation bias. Single-center data require external validation.
Yamashita et al. 2021 [10]	Clinical cohort of 40 patients undergoing EUS; evaluated CP and pancreatic exocrine/endocrine dysfunction.	EUS-SWM; point/box-based shear-wave measurement. Vendor/software details NR in the original summary table.	Median shear-wave velocity used. ROI site, number of measurements, breath control, and quality criteria should be explicitly specified if available from full text.	Japan Pancreatic Society criteria for CP; pancreatic exocrine and endocrine dysfunction as functional endpoints.	AUROC: CP 0.92, exocrine dysfunction 0.78, endocrine dysfunction 0.63. Cut-offs: 1.96 m/s for CP, 1.96 m/s for exocrine dysfunction, 2.34 m/s for endocrine dysfunction. Sensitivity/specificity: 83%/100%, 90%/65%, and 75%/64%, respectively.	Specific valid acquisition rate and inter-/intra-observer reproducibility NR in the manuscript table.	Suggests association between stiffness and functional impairment. However, small sample size, JPSC/EUS-related reference criteria, and absence of external validation limit immediate clinical translation.
Yamashita et al. 2023 [11]	Prospective comparative study; 49 patients evaluated with CT, EUS-SWE/SWM, strain elastography, and pancreatic exocrine function testing.	EUS shear-wave technique compared with strain elastography; acquisition mode should be reported as SWM/point SWE if no 2D stiffness map was used.	Acquisition protocol and quality-control criteria NR in the current table; these should be retrieved from full text and added where available.	Multiple reference standards: CT findings, Rosemont criteria, Japan Pancreatic Society 2019 criteria, and pancreatic exocrine dysfunction.	EUS shear-wave technique outperformed strain elastography across standards: AUROC CT 0.77 vs. 0.61, Rosemont 0.85 vs. 0.56, JPSC 0.83 vs. 0.53, exocrine dysfunction 0.78 vs. 0.61.	Specific valid acquisition rate and reproducibility metrics NR in the current table.	Supports SWE/SWM over strain elastography for CP assessment, but use of EUS-based diagnostic standards may inflate diagnostic performance. Cut-offs and external reproducibility should be validated.
Shintani et al. 2025 [12]	Retrospective propensity-score-matched study; early CP versus matched controls after exclusions from a larger EUS-SWE cohort.	EUS-SWE/SWM reported as shear-wave velocity; exact acquisition mode and platform should be specified in the manuscript if available.	Pancreatic body measurements. Reliability assessed with VsN; practical VsN ≥ 50% considered significant/reliable.	Japanese diagnostic criteria 2019 for early CP.	After matching: 22 early CP and 22 controls. Velocity higher in early CP: 2.31 +/− 0.67 vs. 1.59 +/− 0.40 m/s. AUROC 0.82; proposed cut-off 2.24 m/s. Sensitivity/specificity NR in the available summary.	Valid acquisition rate and reproducibility metrics NR in the current table.	Potential objective adjunct for early CP. Interpretation should be cautious because early CP criteria include EUS parenchymal/ductal features; no independent histological or longitudinal outcome standard.
Ikemura et al. 2025 [13]	Retrospective single-center diagnostic accuracy study; 38 patients evaluated for early CP using 2019 Japanese criteria.	EUS-SWM; point/box-based shear-wave measurement. Vendor/software details should be reported if available from full text.	Protocol details NR in the current table. Final manuscript should specify ROI location, number of measurements, breath control, valid measurement criteria, and failed measurement handling.	2019 Japan Pancreatic Society early CP criteria; patients categorized according to number of positive EUS findings.	EUS-SWM higher in patients with early CP findings: 2.39 +/− 0.54 vs. 1.84 +/− 0.46 m/s. AUROC 0.78; cut-off 1.87 m/s; sensitivity 90.9%, specificity 63.0%.	Valid acquisition rate and reproducibility metrics NR in the current table.	Small retrospective study. Reference standard is related to EUS morphological changes, creating possible incorporation bias. Requires prospective external validation and outcome-based confirmation.
Ohno et al. 2019 [15]	Prospective exploratory study of AIP activity; 160 patients included, with comparison between AIP and normal pancreas and a small post-steroid reassessment subgroup.	EUS-SWM using linear-array echoendoscope GF-UCT260 and ARIETTA 850 ultrasound system; point/box-based measurement, not true 2D-SWE.	Measurements in pancreatic head, body, and tail. Body/tail from stomach; head from duodenum. Rectangular ROI approximately 5 x 10 mm, placed 5–10 mm below probe while avoiding vessels, ducts, and cystic areas. Five reliable measurements targeted; VsN >= 50% used for reliability.	Clinical diagnosis of AIP and clinical/steroid response; histological confirmation not uniformly specified in the summary.	AIP showed higher shear-wave velocity than normal pancreas: body median 2.57 vs. 1.89 m/s. In six patients reassessed after corticosteroids, mean velocity decreased from 3.32 to 2.46 m/s.	3837 measurements; successful measurement rate 97.6%. No adverse events reported.	Biologically plausible marker of inflammatory activity and response. Evidence limited by small AIP subgroup, very small longitudinal steroid-response sample, and absence of validated cut-offs for activity, relapse, or remission.
Ohno et al. 2021 [16]	Retrospective comparative study of 64 solid pancreatic lesions; compared EUS-SWM with strain elastography and strain histogram analysis.	EUS-SWM for lesion velocity and VsN; strain elastography/strain histogram as comparator. Platform details NR in the current table.	Lesion ROI and Vs/VsN measured in target lesion. Detailed ROI placement, number of measurements, and failed acquisition handling should be extracted from full text if available.	Histology or clinical follow-up. The proportion confirmed by histology versus follow-up is not specified in the current table and should be clarified if available.	Mean Vs did not significantly discriminate pancreatic cancer, PanNEN, mass-forming pancreatitis, and metastasis. Pancreatic cancer vs. mass-forming pancreatitis: Vs difference not significant. Strain histogram performed better than EUS-SWM for PC vs. MFP.	Valid acquisition rate and reproducibility metrics NR in the current table.	Important negative study for standalone diagnosis of solid pancreatic lesions. Results support caution: absolute stiffness overlaps across malignancy, inflammation, and background parenchyma.
Kojima et al. 2025 [17]	Pancreatobiliary cohort including pancreatic cancer; evaluated novel EUS scope/ultrasonographic system and normalized shear-wave parameters.	EUS-SWE/SWM with normalized shear-wave parameter; exact platform, software, and acquisition mode should be explicitly specified from full text.	Absolute velocity and normalized velocity parameters assessed. ROI location, number of acquisitions, and valid acquisition rules NR in the current table.	Composite clinical diagnosis; the current manuscript summary does not clarify whether diagnosis was based on histology, imaging, follow-up, or combined criteria.	Absolute shear-wave velocity overlapped among pancreatic cancer lesions, adjacent/background parenchyma, and normal pancreas. Normalized velocity appeared to improve discrimination, with a proposed normalized threshold around 50%.	Specific technical success, valid acquisition rate, and reproducibility metrics NR in the current table.	Potential value of relative/normalized metrics, but reference standard ambiguity and lack of external validation preclude clinical adoption. The final table should clarify histology/follow-up proportions if available.
Miutescu et al. 2025 [8]	Prospective single-center reproducibility study of pancreatic EUS-guided point SWE in 86 consecutive patients.	EUS-guided point SWE; vendor/software details should be checked in full text before final insertion.	Ten measurements from a 10 x 15 mm ROI in pancreatic body or tail during breath-hold by a single expert operator. VsN > 50% used as reliability criterion.	Technical reproducibility and intra-individual variability; not a diagnostic accuracy study.	Mean stiffness 18.5 +/− 8.9 kPa, corresponding to 2.31 +/− 0.58 m/s.	Technical success 97%; no adverse events. ICC for early vs. late measurements: 0.99 in kPa but 0.61 in m/s, highlighting unit-dependent variability.	Useful for the reviewer-requested reproducibility discussion. Single-center/single-operator design and lack of disease-specific diagnostic validation limit generalizability.

Abbreviations: AIP, autoimmune pancreatitis; AUROC, area under the receiver operating characteristic curve; CP, chronic pancreatitis; EUS, endoscopic ultrasound; EUS-SWE, EUS-guided shear-wave elastography; EUS-SWM, EUS-guided shear-wave measurement; JPSC, Japan Pancreatic Society criteria; MFP, mass-forming pancreatitis; NR, not reported in the manuscript table or accessible abstract-level summary used for revision; PanNEN, pancreatic neuroendocrine neoplasm; PC, pancreatic cancer; QC, quality control; ROI, region of interest; SWE, shear-wave elastography; Vs, shear-wave velocity; VsN, net effective shear-wave velocity percentage. Clinical interpretation note: diagnostic performance in CP and early CP studies should be interpreted with caution because reference standards such as Rosemont and JPSC criteria include EUS parenchymal or ductal features that may be mechanistically related to pancreatic stiffness.

**Table 3:** Liver, spleen, and endo-hepatology applications of shear-wave elastography relevant to EUS-guided shear-wave-based elastography.

Study, Reference	Design and Population	EUS Platform and Acquisition Mode	ROI, Acquisition Protocol and Quality Control	Reference Standard	Main Quantitative Findings	Feasibility and Reproducibility	Major Limitations and Clinical Interpretation
Cassinotto et al. 2020 [21]	Patients with chronic liver disease; transabdominal liver stiffness study used as clinical benchmark for 2D-SWE rather than as an EUS study.	Transabdominal 2D-SWE; not EUS-guided. Vendor/platform should be specified if retained in the final table.	Transabdominal liver stiffness measurements. EUS-specific route, scope position, sedation, and endoscopic workflow are not applicable.	VCTE categories/Baveno-style liver stiffness framework; not histological confirmation for all patients.	2D-SWE cut-off around 10 kPa identified patients with VCTE <10 kPa with high sensitivity and specificity. AUROC reported as excellent for VCTE-based categories.	Reproducibility/valid acquisition data NR in te current table.	Useful comparator for liver stiffness thresholds, but should be labelled as a non-EUS benchmark. It should not be used as direct evidence for EUS-SWE clinical readiness.
Kohli et al. 2023 [22]	Single-center prospective non-randomized tandem study of patients undergoing liver biopsy; compared EUS-SWE with VCTE and histology.	EUS-SWE liver stiffness measurement; vendor/software details NR in the current table and should be added if available.	At least 10 elasticity values obtained; reliability required VsN >= 60% for each measurement, with final median value used. Lobe-specific measurements considered.	Liver histology and VCTE comparator.	EUS-SWE correlated with histology and showed accuracy comparable to VCTE. VCTE was unreliable in 8/42 patients, whereas EUS-SWE was technically successful in all patients in the reported cohort.	Technical success 100% in the reported cohort. Detailed interobserver reproducibility NR in the current table.	Promising, especially when VCTE is unreliable, but study was small and single-center. EUS-SWE requires endoscopy, sedation, procedural resources, and should not be framed as non-invasive.
Diehl et al. 2025 [23]	Prospective pilot reproducibility cohort; 52 patients underwent TE, EUS-SWE of both liver lobes, and liver biopsy.	EUS-SWE liver stiffness; TE comparator. Platform/software details NR in the current table.	Right- and left-lobe EUS-SWE measurements obtained; paired consecutive measurements used to assess variability. Exact route, scope position, number of acquisitions, and sedation details should be specified from full text.	Liver biopsy histology using METAVIR; TE comparator.	Right-lobe EUS-SWE correlated more strongly with fibrosis stage than left-lobe EUS-SWE. Left-lobe paired measurements showed substantially higher variance. Discrimination for fibrosis was similar to TE in this pilot cohort.	Key reproducibility finding: left-lobe measurements showed approximately 3.5-fold higher variance between consecutive measurements than right-lobe measurements.	Highlights importance of lobe selection and acquisition standardization. Limitations include small sample size, pilot design, limited advanced fibrosis spectrum, and need for validated cut-offs.
Wang et al. 2025 [24]	Multicenter cross-sectional pilot study; patients with obesity and suspected MASLD/MASH underwent EUS-SWE and liver biopsy; compared with FIB-4 and VCTE.	EUS-SWE liver stiffness; platform/software details should be specified in final table if available.	EUS-guided liver stiffness acquisition; exact lobe, route, number of acquisitions, ROI depth, and quality-control criteria should be reported from full text.	Liver biopsy histology; comparison with FIB-4 and VCTE.	In 62 patients with mean BMI about 40.7 kg/m^2^, AUROC for EUS-SWE was high for significant fibrosis, advanced fibrosis, and cirrhosis. Reported 90% sensitivity cut-offs: F2 7.50 kPa, F3 8.48 kPa, F4 11.30 kPa. Reported 90% specificity cut-offs: F2 9.82 kPa, F3 10.20 kPa, F4 14.60 kPa.	Technical success and reproducibility metrics NR in the current table.	Potential niche in obesity/MASLD when transabdominal tests are unreliable. However, this remains a pilot, selected-population study; EUS invasiveness, sedation, cost, and endoscopy-unit burden must be weighed against non-invasive tests.
AbiMansour et al. 2025 [25]	Prospective cohort/real-world advanced liver disease assessment; 274-patient series reported in current sources.	EUS-SWE liver stiffness. Device/platform, software, and acquisition mode details NR in the current table and should be checked in full text.	Lobe-specific EUS-SWE measurements reported; exact ROI, route, number of measurements, valid acquisition criteria, and handling of failed measurements should be added if available.	MRI elastography/clinical fibrosis assessment. The prior wording “clinical and fibrosis assessment” is too vague and should be replaced by the explicit comparator/reference standard used in the article.	Reported AUROC for >=F3 fibrosis approximately 0.73 for left lobe and 0.80 for right lobe. Increasing EUS-SWE values were associated with increasing MRE stiffness.	Specific valid acquisition and reproducibility metrics NR in the current table.	Provides supportive real-world data but diagnostic performance is moderate and reference standard is not uniformly histology.
Yousaf et al. 2025 [26]	Single-center endo-hepatology experience combining EUS-SWE, EUS-guided portal pressure gradient measurement, and EUS-guided liver biopsy.	Integrated EUS-based platform: EUS-SWE/SWM plus EUS-PPG and EUS-guided liver biopsy. Platform/software details NR in the current table.	Combined procedure; acquisition route, lobe selection, number of stiffness measurements, PPG protocol, sedation, procedure time, and adverse events should be specified if available.	Liver biopsy histology and direct EUS-guided portal pressure gradient measurement.	High technical success reported in the manuscript summary. EUS-SWE/SWM and EUS-PPG correlated with fibrosis stage in the integrated endo-hepatology setting. Exact AUROC/cut-offs NR in the current table.	Detailed reproducibility metrics and valid acquisition rates NR in the current table.	Conceptually important one-step endo-hepatology model, but not equivalent to routine non-invasive fibrosis assessment. Requires endoscopy, sedation, procedural time, expertise, cost, and dedicated workflow.
Furuichi et al. 2023 [27]	Study of splenic stiffness/dispersion slope for portal hypertension or esophageal varices; included as physiological rationale for splenic stiffness assessment.	Splenic SWE/dispersion slope; transabdominal or non-EUS approach in the available summary. Not direct EUS-SWE evidence unless full text specifies otherwise.	Splenic stiffness acquisition details NR in the current table. EUS-specific scope position, route, and sedation are not established from the summary.	Esophageal varices/portal hypertension assessment, presumably by endoscopy and clinical criteria; exact reference standard should be clarified from full text.	Splenic stiffness metrics evaluated for prediction of esophageal varices. Exact cut-offs, AUROC, sensitivity, and specificity NR in the current table.	Reproducibility/valid acquisition metrics NR.	Should be retained only as background rationale for splenic stiffness and portal hypertension research, not as evidence that EUS-SWE is ready for routine portal hypertension risk stratification.

Abbreviations: AUROC, area under the receiver operating characteristic curve; BMI, body mass index; EUS, endoscopic ultrasound; EUS-PPG, EUS-guided portal pressure gradient; EUS-SWE, EUS-guided shear-wave elastography; FIB-4, fibrosis-4 index; MASLD, metabolic dysfunction-associated steatotic liver disease; MASH, metabolic dysfunction-associated steatohepatitis; MRE, magnetic resonance elastography; NR, not reported in the manuscript table or accessible abstract-level summary used for revision; QC, quality control; ROI, region of interest; SWE, shear-wave elastography; TE, transient elastography; VCTE, vibration-controlled transient elastography; VsN, net effective shear-wave velocity percentage. Interpretative note: transabdominal studies are included only as comparator or physiological benchmark data and should be clearly separated from direct EUS-guided evidence.

## Data Availability

No new data were created or analyzed in this study. Data sharing is not applicable to this article.

## Supplementary Materials

The following supporting information can be downloaded at: https://www.mdpi.com/article/10.3390/diagnostics16162505/s1, Table S1: Comparative clinical positioning of EUS-guided shear-wave-based elastography relative to established diagnostic modalities.

## Abbreviations

The following abbreviations are used in this manuscript:

2D-SWE	Two-dimensional shear wave elastography
AIP	Autoimmune pancreatitis
ARFI	Acoustic radiation force impulse
AUROC	Area under the receiver operating characteristic curve
BT-PABA	N-benzoyl-L-tyrosyl-p-aminobenzoic acid
CI	Confidence interval
CP	Chronic pancreatitis
CT	Computed tomography
E	Young’s modulus
ECP	Early chronic pancreatitis
EUS	Endoscopic ultrasound / Endoscopic ultrasonography
EUS-PPG	Endoscopic ultrasound-guided portal pressure gradient
EUS-SWE	Endoscopic ultrasound-guided shear wave elastography
EUS-SWM	Endoscopic ultrasound-guided shear wave measurement
FIB-4	Fibrosis-4 index
IQR	Interquartile range
JPSC	Japanese Pancreas Society criteria
kPa	Kilopascal
MASLD	Metabolic dysfunction-associated steatotic liver disease
MR	Magnetic resonance
MRI	Magnetic resonance imaging
pNEN	Pancreatic neuroendocrine neoplasm
PDAC	Pancreatic ductal adenocarcinoma
PPG	Portal pressure gradient
pSWE	Point shear wave elastography
RC	Rosemont classification
ROI	Region of interest
SE	Strain elastography
SD	Standard deviation
SWE	Shear wave elastography
SWM	Shear wave measurement
VCTE	Vibration-controlled transient elastography
Vs	Shear wave velocity
VsN	Normalized shear wave velocity/net effective shear wave velocity
