# Supplementary material for: EUS-Guided Shear-Wave-Based Elastography: Current Evidence and the Emerging Role of Two-Dimensional Shear-Wave Elastography
Source: Diagnostics (Basel). 2026 Aug 8;16(16):2505. doi: 10.3390/diagnostics16162505 (PMC13511822; doi:10.3390/diagnostics16162505)
Supplement: Supplementary file 1 [file diagnostics-16-02505-s001.zip › diagnostics-4365855-supplementary.pdf]

Supplementary Table S1. Comparative clinical positioning of EUS-guided shear-wave-based elastography relative to established diagnostic modalities.

Clinical Setting	Existing standard	Potential Added Value of EUS-SWE	Current Limitation
Chronic pancreatitis	Morphological EUS, CT/MRI, and function testing	Quantitative assessment of diffuse stiffness; possible longitudinal biomarker	Incorporation bias; non-validated cut-offs
Early chronic pancreatitis	Clinical criteria, EUS morphology, and function testing	Possible objective adjunct in equivocal disease	Small cohorts; uncertain natural-history correlation
Autoimmune pancreatitis	Clinical, serological and imaging criteria	Potential monitoring of inflammatory response	Very limited longitudinal evidence
Solid pancreatic lesions	Tissue acquisition, CE-EUS, and CT/MRI	Multiparametric characterization	Stiffness overlap; cannot replace histology
Liver fibrosis	VCTE, transabdominal SWE, serum scores, and MRE	Measurement during an already-indicated EUS; possible use when transabdominal tests fail	Invasive, resource-intensive, and limited validation
Endo-hepatology	Biopsy and portal pressure assessment	One-session multiparametric evaluation	Procedural burden and uncertain incremental outcome benefit

Abbreviations: CE-EUS, contrast-enhanced endoscopic ultrasound; CT, computed tomography; EUS, endoscopic ultrasound; EUS-SWE, EUS-guided shear-wave elastography; MRE, magnetic resonance elastography; MRI, magnetic resonance imaging; pSWE, point shear-wave elastography; SWE, shear-wave elastography; SWM, shear-wave measurement; VCTE, vibration-controlled transient elastography.

Footnote: This table summarizes the potential clinical role of EUS-guided shear-wave-based elastography in relation to currently established diagnostic pathways. The proposed added value should be interpreted as hypothesis-generating because most available evidence derives from EUS-guided shear-wave measurement or point shear-wave elastography rather than true two-dimensional shear-wave elastography. EUS-guided shear-wave techniques should currently be considered adjunctive rather than replacement tests. Their use appears most plausible in selected patients already undergoing EUS, particularly when quantitative tissue characterization may complement conventional imaging, tissue acquisition, pancreatic function assessment, liver biopsy, or portal pressure measurement. Reported stiffness values and diagnostic cut-offs are generally platform-, software-, acquisition-mode-, anatomical-site-, and protocol-specific and should not be extrapolated across systems without dedicated validation.
